# Polystyrene Attached Pt(IV)–Azomethine, Synthesis and Immobilization of Glucose Oxidase Enzyme

**DOI:** 10.3390/ijms130911870

**Published:** 2012-09-19

**Authors:** Nurşen Sarı, Esin Antepli, Dilek Nartop, Nurdan Kurnaz Yetim

**Affiliations:** 1 Department of Chemistry, Faculty of Science, Gazi University, Ankara 06500, Turkey; E-Mails: esinantpl@hotmail.com (E.A.); nurdankurnaz@gazi.edu.tr (N.K.Y.); 2 Department of Chemistry, Faculty of Arts and Science, Nevşehir University, Nevşehir 50300, Turkey; E-Mail: dileknartop@nevsehir.edu.tr; 3 Department of Chemistry, Faculty of Science, Kırklareli University, Kırklareli 39999, Turkey

**Keywords:** Pt(IV)—azomethine, Polystyrene, glucose oxidase enzyme, two optimum pH

## Abstract

Modified polystyrene with Pt(IV)–azomethine (APS–Sch–Pt) was synthesized by means of condensation and demonstrated to be a promising enzyme support by studying the enzymatic properties of glucose oxidase enzyme (GOx) immobilized on it. The characteristics of the immobilized glucose oxidase (APS–Sch–Pt–GOx) enzyme showed two optimum pH values that were pH = 4.0 and pH = 7. The insertion of stable Pt(IV)–azomethine spacers between the polystyrene backbone and the immobilized GOx, (APS–Sch–Pt–GOx), increases the enzymes’ activity and improves their affinity towards the substrate even at pH = 4. The influence of temperature, reusability and storage capacity on the free and immobilized glucose oxidase enzyme was investigated. The storage stability of the immobilized glucose oxidase was shown to be eleven months in dry conditions at +4 °C.

## 1. Introduction

Enzymes are used as biocatalysts in the chemical, pharmaceutical and food industries and as specific ligands in clinical and chemical analyses [1]. Glucose oxidase (GOx) is a commercially important enzyme, which has applications in the pharmaceutical industry as a biosensor for the enzymatic determination of glucose in the fermentation of liquor, and in the food industry for the removal of glucose and shelf life of various products [2,3]. Although glucose oxidase enzyme has attracted interest in the varying processes, this enzyme is unstable due to its complex molecular structure. Therefore, a number of immobilization techniques have recently been investigated to improve its stability [4]. To date, different methods have been used for enzyme immobilization [5–8]. One of the most useful immobilization methods is covalent attachment of the enzyme onto a solid support. Generally, there are two types of covalent attachment strategies. One is one point covalent attachment; the other is multipoint covalent attachment. In the one point covalent attachment, only one (or two) functional groups of the enzyme is (are) connected to the polymer support via covalent binding. In the multipoint case, all parts of the enzyme can be connected to the solid carrier via covalent bonds [5].

The chemistry of polymer-based macromolecules has been receiving significant attention as one of the promising immobilization alternatives for biopolymer biosensors in various industry areas. Furthermore, the coordinating ability of the metal within the polymer-based macromolecules permits these materials to act as sensors [9]. Although studies on enzyme immobilization of the polymer-based macromolecules including metal atoms (Si, Se, *etc*.) on transition metal does not show this. In fact, polymer attached metals which have a coordination capability may bond with –NH_2_ and –COOH groups of the enzyme [10]. Of course, the active center of the enzyme must be protected during the formation of this bond. GOx is covered with carbohydrate chains. A carbohydrate chain has hydroxyl groups. Hydroxyl groups can act as good chelating agents with metal ions as coordination covalent bonds. Here, we developed a strategy to immobilize GOx by using polymeric supports with Pt(IV) ions which were prepared from (aminomethyl)polystyrene, 2-hydroxybenzaldehyde and Pt(IV) by template method.

**Scheme 1:**
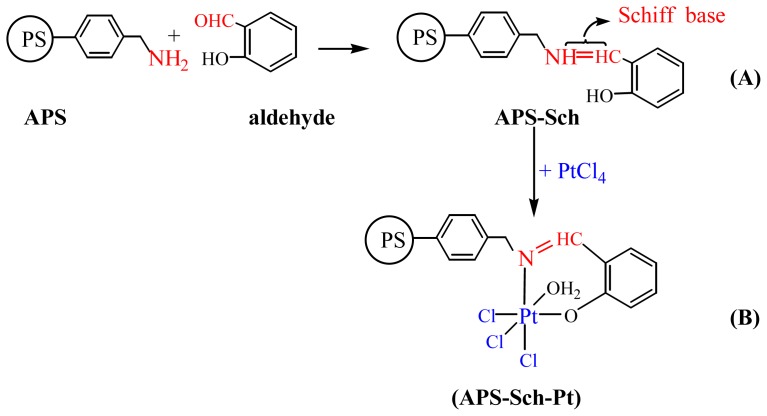
Synthesis route of support for immobilization of glucose oxidase enzyme (GOx) enzyme.

To investigate to what extent the effect of coordination covalent bonds depends on enzyme immobilization, we prepared a polymeric support involving Pt(IV) ions. To prepare such a polymeric support, the (aminomethyl) polystyrene (APS) reacted with 2-hydroxybenzaldehyde (APS–Sch; Scheme 1A) and then the metal-containing complex was prepared using PtCl_4_ salts (APS–Sch–Pt), (Scheme 1B).

## 2. Results and Discussion

### 2.1. Characterization Studies

Analytical data and some of the physical properties of all (APS–Sch–Pt) are given in Table 1. The elemental analyses can be considered compatible with the chemical formulas of the compounds due to polymers of different chain lengths [11]. The average molecular weight (*M*_w_) was estimated from element analyses.

IR Spectra of Polymer-Bound Azomethine–Pt(IV) Complex (APS–Sch–Pt)

The characteristic peak of the IR spectra of the (APS–Sch–Pt) support polymer is given in Table 1. Three overtone peaks are shown at 1943, 1873 and 1800 cm^−1^ of the (APS–Sch–Pt) support polymer. IR bands in the 3433, 3013, 2920, 556 and 541 cm^−1^ regions are characteristic of *ν*(OH) (for H_2_O in Pt(IV) complexes), *ν*(CH) aromatic, *ν*(CH) aliphatic, *ν*(CH) buckling out of plane and *ν*(CH) buckling, respectively. An imine band was observed at 1634 cm^−1^. Also, new weak bands appeared in the 464 and 532 cm^−1^ region, belonging to *ν*(Pt–N) and *ν*(Pt–O), respectively. This confirmed the formation of a coordinate covalent bond consisting of one nitrogen and phenol oxygen for all modified complexes [12]. Measurement of magnetic susceptibility of the metal-containing azomethine polymer is given in Table 1. The Pt(IV) complex of the polymer-bound azomethine polymer found to be diamagnetic. This is the reason why it is suggested that the Pt(IV) complex is octahedral [13].

### 2.2. Immobilization Studies

#### 2.2.1. Influence of pH on the Enzyme Activity

**Figure 1:**
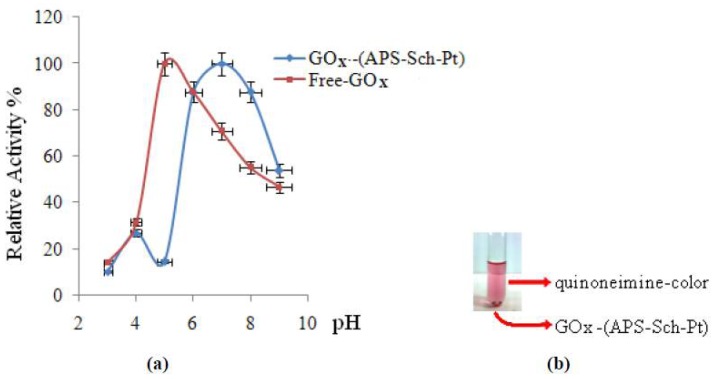
Effect of pH on enzyme activity (**a**) and image of GOx–(APS–Sch–Pt) at optimum pH (**b**).

The maximum activity was obtained at pH 5.0 for the free enzyme. Studying (APS–Sch–Pt), two maximum activities were observed at pH 4.0 and pH 7.0, which is different from earlier reports [14–16]. They are illustrated in Figure 1 and Table 2. As is known, pH is one of the important parameters capable of altering enzymatic activities in aqueous solution. Immobilization of the enzyme is likely to result in conformational changes of the enzyme resulting in a variation of the optimum pH. The reason for having two optima of immobilized (APS–Sch–Pt) may be due to the active properties of different residues of enzyme.

The active site of the enzyme contains three amino acid side chains that are intimately involved in catalysis: His516 with p*K*_a_ = 6.9 and Glu412 with p*K*_a_ = 3.4 which is hydrogen bonded to His559, with p*K*_a_ > 8. The protonation of each of these residues has a strong influence on all rate constants in the catalytic mechanism [15,17]. For an optimum pH = 4, we can say that Glu412 may be more effective than His516 and His559 in the catalytic mechanism. The optimum pH for the effective catalytic mechanism of His516 contained in the enzyme is seven. To our knowledge, two optimum states are not a common property.

#### 2.2.2. Influence of Temperature on the Enzyme Activity

**Figure 2:**
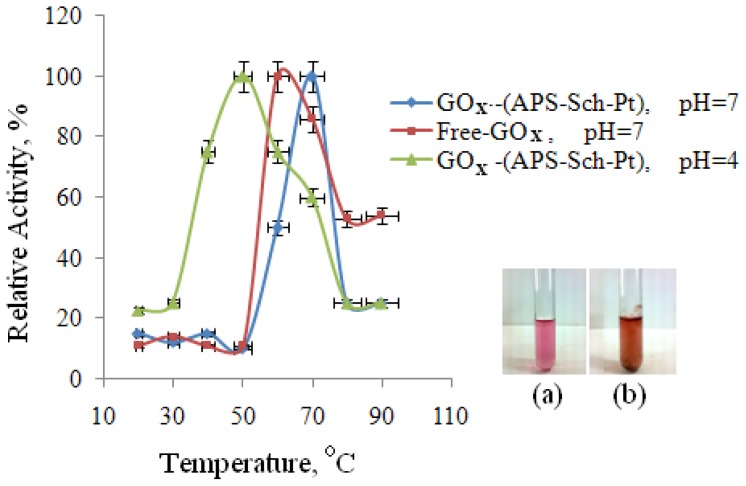
Effect of temperature on enzyme activity and image of GOx–(APS–Sch–Pt) (**a**) and free-GOx at optimum temperature (**b**).

The effect of temperature on the activity of free and immobilized glucose oxidase is shown in Figure 2. There is a significant difference between the profiles of the temperature optimum for free and immobilized GOx. The optimum temperature for free GOx in pH = 4 is not shown due to denatured (Figure 2b). But, surprisingly, the optimum temperature for immobilized glucose oxidase was found to be 40 °C at pH = 4. Also, at pH = 7, the free and immobilized enzyme activities depended on temperatures in a range of 20 °C–90 °C, indicating that the optimal temperature for attaining the highest activities of free and immobilized GOx was 60 °C and 70 °C, respectively (Table 2). The results show that the immobilized enzyme, especially in a coordination covalently bound system, becomes more stable against heat and denaturing agents.

#### 2.2.3. Storage Stability and Reusability of Immobilized Enzyme

**Figure 3:**
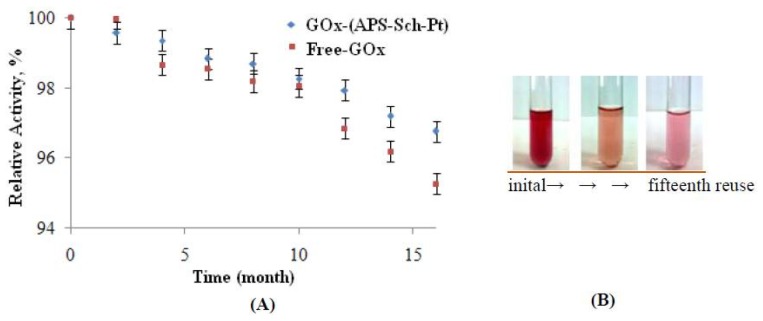
Effect of reuse of immobilized enzyme (**A**); Reuse image of activity of initial and fifteenth use of immobilized enzyme (**B**) at pH = 7 and *t* =70 °C.

The storage stability of an enzyme is one of its most important characteristics. Enzymes often lose their activity during storage. Free and immobilized enzymes were stored in a dark bottle at +4 °C for 16 months (Figure 3) in a dry form. After 16 months, the free GOx and immobilized enzyme retained 95.26% and 96.76% of their original activity, respectively. High activitiy was found for the modified polymer. This result suggests that the modified polymer-support confers a higher conformational stability on the immobilized enzyme due to the formation of coordinate covalent bonds with the Pt(IV) atom (Figure 3).

The reusabilty was tested because of its importance for repeated applications in a batch reactor. Reusability is advantageous for cost-reduction of the treatment [8,18]. The immobilized polymer was used repeatedly four times a day due to long incubation times. After the fifteenth use of (GOx–APS–Sch–Pt), the immobilized enzyme retained nearly 78% of its original activity at pH = 7 and *t* = 70 °C. Our results indicate that the covalent binding of GOx using Pt(IV)–Schiff base helped the enzyme to retain catalytic activity when it was repeatedly used in various cycles. The free enzyme does not show any activity at pH = 4 and *t* = 40 °C due to denaturation (Figure 2b). This result is consistent with the literature. After the sixth use of the immobilized enzyme nearly 23% of its original activity at pH = 4 was retained. This result is an innovation for the immobilization application.

#### 2.2.4. Kinetic Parameters

**Figure 4:**
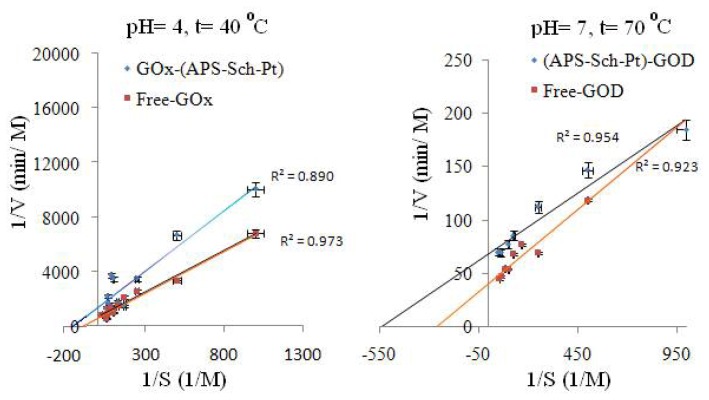
Lineweaver-Burk plots for free and immobilized GOx at pH = 4 and pH = 7.

The activities of the free and immobilized enzymes with various substrate concentrations were plotted as Lineweaver-Burk graphs to calculate *V*_max_ and *K*_m_ values (Figure 4, Table 3). The *V*_max_ value defines the maximum velocity when all of the enzyme is saturated with substrate. *K*_m_, the substrate concentration at which an enzyme reaches ½ *V*_max_, reflects the effective characteristic of the enzyme and depends on both partitioning and diffusion [3].

Kinetic parameters were studied for free GOx at pH = 4 and *t* = 40 °C, because the optimum for immobilized glucose oxidase (at *t* = 40 °C) was found to be at pH = 4. The effect of the substrate concentration on the reaction rate was studied using varying initial concentrations (2–40 mM) of β-D-glucose substrate. The Michaelis-Menten constant (*K*_m_) and the maximum reaction rate (*V*_max_) of free and immobilized GOx were calculated from the Lineweaver-Burk plots (Figure 4). The *K*_m_/*V*_max_ values were calculated to be 0.013 M/0.0016 mmin^−1^ for free GOx and 0.00863 M/0.00069 mmin^−1^ for immobilized GOx to the (APS–Sch–Pt) supports, respectively.

For optimum pH = 7 and *t* = 70 °C;

As mentioned above, kinetic parameters were studied with the same concentrations of substrate. *K*_m_ and *V*_max_ were calculated to be 0.00377 M/0.0245 Mmin^−1^ for free GOx and 0.0018472 M/0.01466 Mmin^−1^ for immobilized GOx to the (APS–Sch–Pt) supports, respectively.

#### 2.2.5. Leaching Study

No marked decrease of the effect of medium washing on immobilized glucose oxidase activity was observed. This may be a result of the strong coordination covalent bond between Pt(IV) and the hydroxyl group of the enzyme. Furthermore, dry immobilized glucose oxidase activity was not denatured during working time (of two or three days).

## 3. Experimental Section

### 3.1. Chemicals

Glucose oxidase (β-d-glucose: oxygen-l-oxidoreductase, EC 1.1.3.4) from *Aspergillus niger* was purchased from Sigma Chemical Company (SIGMA, 49180). Its molecular weight and pI was 160,000 Da and 4.2, respectively. 4-aminoantipyrene (4-AAP), phenol, (aminomethyl)polystyrene, 2-hydroxy-5-benzaldehyde, and PtCl_4_ were purchased from Sigma (St. Louis, MO, USA). All the other chemicals used in this work were provided by Merck and Sigma-Aldrich and used without further purification.

### 3.2. Apparatus for Characterization

Carbon, hydrogen and nitrogen analyses were carried out with an Elementary Micro Vario CHNS instrument. Platinum contents were determined by using a Philips PU 9285 atomic absorption instrument at Metu-central lab, Ankara, Turkey. IR spectra were recorded on a Mattson-5000 FT-IR instrument in KBr pellets.

### 3.3. Synthesis of Support Polymer (APS–Sch–Pt)

Pt(IV)-containing support polymer was synthesized by following a general method. The polymeric-azomethine (APS-Sch) was prepared by reacting (aminomethyl) polystyrene (APS) (1 g, 100–200 mesh, 0.5–1.0 mmol/g –NH_2_ loaded 1% cross-linked (Aldrich)) in hot DMF (15 mL) with aldehyde (2-hydroxy benzaldehyde; 1.0 mmol) in DMF (10 mL) and stirring for 2 h under a reflux condenser at 50 °C. PtCl_4_ (1.0 mmol) in DMF (5 mL) was added to the mixture during 15 min and the mixing process was continued for *ca*. 4 h. Then the Pt(IV)-containing polymer complex was obtained (Scheme 1). After the mixture was cooled to room temperature, the polymer attached Pt(IV) was poured into the acetone and washed by adding acetone. The resulting solid (dark brown) was filtered and dried in an oven.

### 3.4. Immobilization of GOx on (APS–Sch–Pt)

The (APS–Sch–Pt) polymer (0.0125 g) was placed in a 15 mL DMF: water solution (9:6) of 0.010 gL^−1^ of glucose oxidase at 30 °C in a shaking water bath for 2 h. The immobilized polymer was separated and the free enzyme was removed by washing with phosphate buffer (pH: 7, 15 mL). The immobilized enzymes were freshly used and then stored at 4 °C. Saturation ratio (s.o.) was determined as 99.83% from absorbance value in 504 nm. This ratio was calculated by the following formula.

$$\begin{array}{c} A^{504}=ɛ\times b\times C_{8\;\text{mL},0.1\;\text{mg}/\text{mL}}\\ {A_{\text{s}.\text{o}.}}^{504}=ɛ\times b\times C_{\left(8\;\text{mL},\;0.1\;\text{mg}/\text{mL}-\text{immobilized }{\text{GO}}_{\text{x}}\right)} \end{array}$$

### 3.5. Assay for Enzyme Activity Measurement

A colorimetric method based on Trinder’s reaction was used for the determination of the glucose concentration [19]. Glucose is enzymatically oxidized to gluconic acid and hydrogen peroxide in the presence of glucose oxidase. The hydrogen peroxide reacts with 4-aminoantipyrene (4-AAP) and phenol to form a pink colored quinoneimine dye, which has an absorption maximum at 504 nm (*A*_504_). The following reaction was started by adding 16 mg glucose after pre-incubating at 30 °C for 15 min. This mixture was removed after incubating the reaction mixture at 30 °C for 75 m under continues stirring. Then, it was transferred to a quartz cuvette for measurement.

$$\begin{array}{c} \text{glucose}+{\text{H}}_{2}\text{O}+{\text{O}}_{2}\overset{{\text{GO}}_{\text{x}}}{\to }\text{gluconic acid}+{\text{H}}_{2}{\text{O}}_{2}\\ {\text{H}}_{2}{\text{O}}_{2}+4-\text{AAP}+\text{phenol}\overset{\text{peroxidase}}{\to }\text{quinoneimine}+{\text{H}}_{2}\text{O} \end{array}$$

The following recipe was used for the free enzyme/immobilize enzyme assay:

Four milliliters studied buffer (pH 3–9) + 10 mg 4-aminoantipyrene + 20 mg phenol + 0.5 mg of horseradish peroxidase (HRP) + 0.010 g/L, 6 mL free glucose oxidase/immobilize glucose oxidase in studied buffer + 16 mg glucose.

### 3.6. Effect of pH and Temperature on Activity of Free and Immobilized GOx

The optimum pH for free and immobilized glucose oxidase was determined by measuring the activity of free and immobilized enzymes in buffers of different pH values ranging from 3 to 10. The buffers used were: pH: 3–4 (NaH_2_PO_4_/H_3_PO_4_); pH: 5 (sodium acetate/acetic acid); pH: 6 (Na_2_HPO_4_/NaH_2_PO_4_); pH: 7–9 (NaH_2_PO_4_/Na_2_HPO_4_); pH: 10 (Na_2_B_4_O_7_/NaOH).

In the case of temperature studies, free and immobilized enzymes were incubated in the reaction mixtures at different temperatures ranging from 25 °C to 90 °C. The activities of free and immobilized enzyme were plotted against the respective temperature.

### 3.7. Effect of Substrate

#### 3.7.1. For Optimum pH = 4 and 40 °C Temperature

To determine the extent at which immobilization affected the enzyme activity, *K*_m_ and *V*_max_ were determined at optimum pH and 40 °C temperature [20]. Free and immobilized enzyme were incubated with different substrate concentrations (2–40 mM) in phosphate buffer of pH 4 and assayed for enzyme activity at 40 °C, a recommended temperature for enzyme assays.

#### 3.7.2. For Optimum pH = 7 and 70 °C Temperature

To determine the extent at which immobilization affected the enzyme activity, *K*_m_ and *V*_max_ were determined at optimum pH and 70 °C temperature [20]. Free and immobilized enzyme were incubated with different substrate concentrations (2–40 mM) in phosphate buffer of pH 7 and assayed for enzyme activity at 70 °C, a recommended temperature for enzyme assays.

### 3.8. Storage Stability and Reusability of Immobilized Enzyme

Storage stability experiments were carried out to determine the stabilities of immobilized enzymes after storage in dry conditions at +4 °C during eleven months. The enzyme activity was measured every 10 days. Observed results compared to the initial activities. To evaluate the reusability, the glucose oxidase immobilized polymeric supports were also washed with buffer solution after every run and re-introduced into a fresh solution. Reaction cycles were performed under the conditions (pH = 4, *t* = 30 °C; pH = 7, *t* = 70 °C) described above. The enzyme activity was measured every 2 h.

### 3.9. Glucose Oxidase Leaching

The (APS–Sch–Pt)-immobilized glucose oxidizes (0.0125 g) were placed into a sealed tube with H_2_O (4.0 mL). The mixtures were shaken at 30 °C in a shaking water bath for 2 h. The immobilized enzymes were isolated from the studied medium (depending on pH and temperature) by filtration through filter paper. This procedure was repeated accordingly up to three cycles for pH = 4, *t* = 40 °C and pH = 7, *t* = 70 °C. And after, the filtrate was treated according to the procedure described in Section 3.5 and no free enzyme activity was observed.

## 4. Conclusions

**Scheme 2:**
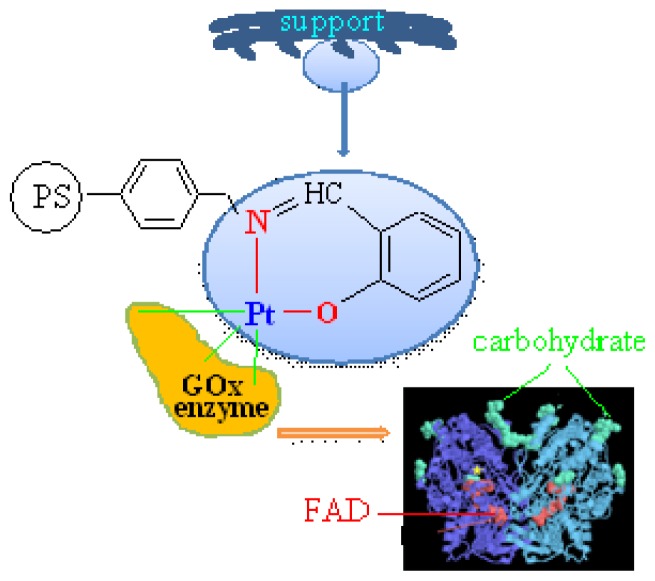
Image of coordination covalent bond between GOx and the Pt(IV) atom.

In this study, we developed a novel strategy for the immobilization of GOx enzyme based on Pt(IV)-Schiff base modified polymeric supports, which are synthesized from condensation of polymer containing –NH_2_ with 2-hydroxybenzaldehyde. As is known, GOx is dimeric protein and is covered with carbohydrate chains. A carbohydrate chain has hydroxyl groups (Scheme 2). These groups can act as good chelating agents for the transition of metal ions. In this study, a coordination covalent bond may have occurred between the Pt(IV) atom and the hydroxyl group of the enzyme. Hence, flavin adenine dinucleotide (FAD) works while preserving its structure.

In this study the maximum activities for immobilized enzyme were found at pH = 4.0 and pH = 7.0. In fact, the shoulder or optimum pH in the acidic area was shown in a few other studies as well [3]. However, we observed that the optimum pH = 4 was significant in this study.

In our study the immobilized form of the enzyme was much more stable than the free enzyme. Furthermore, operational and storage stabilities of GOx were dramatically improved following immobilization. These kinds of superior properties of our developed immobilization strategy would be great advantages for various industrial applications, not only for GOx but also for other enzymes.

## Figures and Tables

**Table 1:** Analytical data, some of the physical properties and important infrared (IR) vibration frequencies (cm^−1^) of (APS–Sch–Pt) polymer.

Important IR vibration frequencies (cm^−1^)		Chemical formula: {-(C_8_H_8_)_11_[(C_16_H_14_NOPtC_l3_H_2_O)]-(C_8_H_8_)_11_}-Elemental analysis; Found (calculated)%				*M*_w_	Color	μeff
			
		C	H	N	Pt			
*ν*(O–H)	3433	84.05	6.39	0.42	6.64	3051.5	Brown	Diamagnetic
*ν*(CH=N)	1634	(81.8)	(6.81)	(0.01)	(6.39)			
*ν*(C–H)_aromatic_	3013		-					
*ν*(C–H)_aliphatic_	2920		-					
*ν*(Pt–O)	464		-					
*ν*(Pt–N)	532		-					

*M*_w_: average molecular weight (values are according to elemental analysis); μ_eff_: Magnetic moment.

**Table 2:** Optimum values of free and immobilized GOx.

Optimum conditions	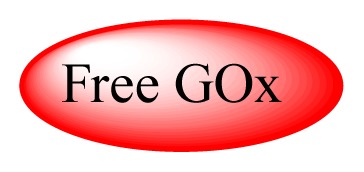	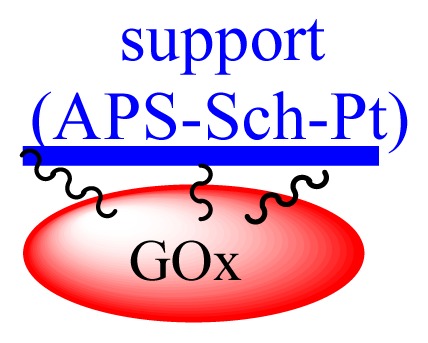	
pH	5	4	7
T (°C)	60	40	70

**Table 3:** Kinetic parameters of free and immobilized GOx.

	*V*_max_ (Mmin^−1^) × 10^−3^		*K*_m_ (M) × 10^−3^	
	
Working conditions	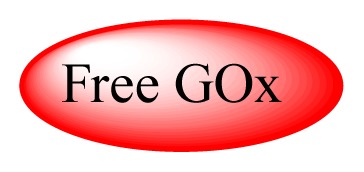	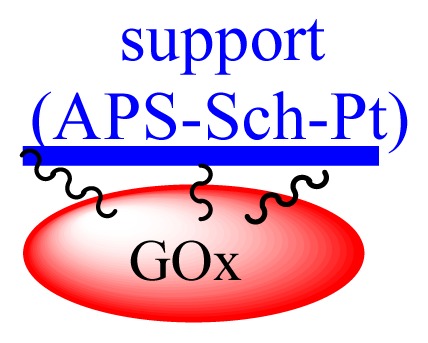	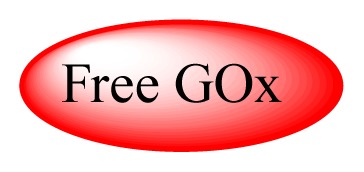	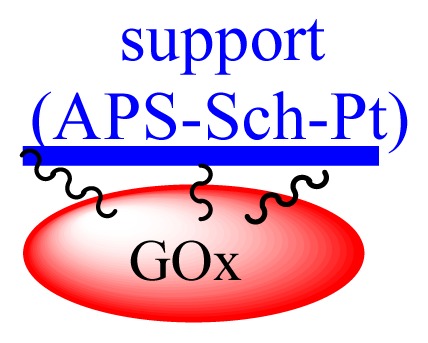
pH = 4, 40 °C	1.6 ± 0.2	0.7 ± 0.2	13.0 ± 0.2	8.6 ± 0.3
pH = 7, 70 °C	24.5 ± 0.1	14.7 ± 0.3	3.8 ± 0.1	1.9 ± 0.2

For optimum pH = 4 and *t* = 40 °C;
